# Between oceans and deserts: fluid balance and outcomes after liver transplantation

**DOI:** 10.1016/j.bjane.2026.844721

**Published:** 2026-01-10

**Authors:** Sergio Henrique Loss, Edino Parolo, Felippe Leopoldo Dexheimer Neto

**Affiliations:** a Hospital de Clínicas de Porto Alegre, Hospital Moinhos de Vento, Porto Alegre, RS, Brazil; b Irmandade Santa Casa de Misericórdia de Porto Alegre, Porto Alegre, RS, Brazil; c Departamento Medicina Interna, Faculdade de Medicina, Universidade Federal do Rio Grande do Sul, Hospital Moinhos de Vento, Porto Alegre, RS, Brazil

After liver transplantation, fluids can damage as much as they can save. In a recent fascicle of the *Brazilian Journal of Anesthesiology*, Lobo et al.^1^ show that both too little and too much fluid are lethal. In a prospective cohort of OLT patients, mortality followed a striking U-shaped curve: those with negative or markedly positive balances fared far worse than those in between. Their message is unambiguous: in transplantation, as in critical care more broadly, both oceans and deserts of fluids are deadly.

The history of intravenous fluid therapy has always swung between extremes. The Swan-Ganz catheter, introduced in 1970,^2^ promised precise hemodynamic guidance. It inspired attempts to achieve supranormal oxygen delivery, often by aggressive resuscitation, but these failed to improve outcomes^3^ and later meta-analyses cast doubt on its benefit.^4^ Decades later, Early Goal-Directed Therapy (EGDT) generated similar enthusiasm after Rivers et al. reported improved survival in septic shock.^5^ Yet when rigorously tested in ProCESS, ARISE, and ProMISe, protocolized EGDT, when tested in multicenter RCTs proved no better than contemporary best practice.^6^, ^7^, ^8^ Once again, rigid formulas collapsed under scrutiny.

Surgery has told the same story. Restrictive strategies were promoted to reduce pulmonary edema, but the RELIEF trial showed that excess restriction caused kidney injury, while liberal regimens increased pulmonary complications.^9^ More recently, de Castro et al.^10^ confirmed that cumulative perioperative balances independently predict pulmonary complications after abdominal surgery. The lesson is simple and consistent: rigid doctrines of fluid therapy — whether liberal or restrictive — carry harm.

Lobo et al.^1^ extend this lesson into liver transplantation. Their prospective study of 73 patients stratified postoperative balances into negative, intermediate, and high. Mortality rates were 18.2%, 8.6%, and 40.5%, respectively. A positive balance on day one was independently associated with death from graft failure. This pattern is not incidental but pathophysiologically coherent. Excess fluids lead to interstitial edema, hepatic congestion, pulmonary dysfunction, renal venous hypertension, and intra-abdominal hypertension.^1^^,^^9^, ^10^, ^11^, ^12^^,^^15^ These mechanisms suffocate the graft and impair recovery. Conversely, inadequate resuscitation starves the graft of blood flow, risking ischemic dysfunction.^1^^,^^5^^,^^8^^,^^10^ As Wise, Nasa, and Malbrain^12^ have argued, “fluid accumulation syndrome” results not only from deliberate resuscitation but also from insidious “fluid creep.” Together, these data explain why both extremes oceans and deserts of fluids harm. Balance is not nuance, it is survival.

These findings align with — rather than prove — broader evidence: in abdominal surgery, de Castro et al.^10^ documented how positive balances drive pulmonary complications, echoing RELIEF.^9^ In sepsis, large trials^5^, ^6^, ^7^, ^8^ and systematic reviews^11^^,^^15^ have shown that excess fluids worsen outcomes. Malbrain and colleagues^15^ emphasized the downstream consequences of edema and intra-abdominal hypertension. Across settings — OLT,^1^ surgery,^9^^,^^10^ sepsis,^5^, ^6^, ^7^, ^8^^,^^11^ and perioperative critical care^12^^,^^15^ — the message converges: oceans and deserts of fluids are equally dangerous.

Likewise important is the identification of the SOFA-liver subscore as an independent predictor of mortality in OLT.^1^ Each one-point increase nearly doubled the risk of death. This reinforces what prior transplant studies demonstrated:^13^^,^^14^ global scores alone are insufficient. Organ-specific monitoring matters. In this population, SOFA-liver is not optional — it is indispensable. It offers a simple, bedside measure of graft function that should be integrated into every postoperative assessment, complementing biochemical markers and hemodynamic indices.^12^, ^13^, ^14^, ^15^

The clinical implications are clear. First, fluids must be prescribed as diagnostic interventions, never as routine maintenance. Each bolus must be justified and reassessed. Second, multimodal monitoring should replace blind reliance on fluid balances. Bedside examination, capillary refill time, ultrasound-derived indices, lactate clearance, and organ scores provide a multidimensional view.^11^, ^12^, ^13^, ^14^, ^15^ Third, timing is critical. Early resuscitation may require positive balances, but persistence of overload by 72 hours — as shown by Lobo et al.^1^ — is a harbinger of death. At that point, clinicians must stop giving and start taking: diuretics and renal replacement therapy should be deployed to evacuate excess.

The four-phase ROSE framework — resuscitation, optimization, stabilization, evacuation — provides a useful model.^12^^,^^15^ In OLT, these phases must progress rapidly. Too often, resuscitation bleeds into days of unnecessary accumulation. As Wise et al.^12^ argue, failure to de-escalate is one of the main drivers of fluid-related harm. Malbrain et al.^15^ remind us that unchecked edema and intra-abdominal hypertension compromise multiple organs. For transplant patients, the cost is even higher: edema and congestion may doom the graft itself.

Critics may point to the limitations of Lobo et al.’s study: single-center design, modest sample, and absolute rather than weight-adjusted balances.^1^ These are valid. But the coherence of their findings with evidence from sepsis,^5^, ^6^, ^7^, ^8^^,^^11^ abdominal surgery,^9^^,^^10^ and perioperative reviews^12^^,^^15^ makes the message compelling. What we need now is not more observational studies but multicenter validation and randomized trials. These should test individualized, physiology-guided strategies, integrating multimodal monitoring, SOFA-liver, and structured de-escalation. The inclusion of bedside tools such as venous congestion ultrasound (VExUS)^12^ could refine assessment of when to evacuate.

The broader story of fluid therapy is one of repeated corrections: from Swan-Ganz optimism^2^, ^3^, ^4^ to EGDT collapse,^5^, ^6^, ^7^, ^8^ from surgical restriction^9^ to recognition of balance.^10^, ^11^, ^12^^,^^15^ Lobo et al.^1^ remind us that in liver transplantation, this lesson is immediate and unforgiving. Their study shows that excess fluids kill, restriction kills, and only balance saves. For clinicians, the practical message is unavoidable: prescribe fluids as carefully as drugs, abandon rigid doctrines, and personalize therapy to phase, physiology, and organ function.

For the scientific community, the challenge is clear. Future trials must abandon the tired question of “restrictive versus liberal.” That debate is obsolete. The real question is how to personalize fluid therapy — to integrate multimodal monitoring, SOFA-liver, and structured de-escalation into everyday practice.

For anesthesiologists and intensivists, this is not optional. The findings of Lobo et al.^1^ demand change. Both oceans and deserts of fluids harm. Balance is not nuance — it is survival.

## Declaration of generative AI and AI-assisted technologies in the manuscript preparation process

During the preparation of this work the author(s) used CHATGPT in order to review language. After using this tool/service, the author(s) reviewed and edited the content as needed and take(s) full responsibility for the content of the published article.

## Author contributions

The authors contributed equally to the conception, elaboration and revision of the manuscript.

## Funding sources

None.

## Conflict of interest

None.
